# Investigating the Mechanisms of Adventitious Root Formation in Semi-Tender Cuttings of *Prunus mume*: Phenotypic, Phytohormone, and Transcriptomic Insights

**DOI:** 10.3390/ijms26062416

**Published:** 2025-03-07

**Authors:** Xiujun Wang, Yue Li, Zihang Li, Xiaowen Gu, Zixu Wang, Xiaotian Qin, Qingwei Li

**Affiliations:** 1 School of Landscape Architecture, Beijing Forestry University, Beijing 100083, China; wangxiujun0520@126.com (X.W.); lynnli0813@126.com (Y.L.); lizihang12318@foxmail.com (Z.L.); dbnydxgxw@163.com (X.G.); zixu.wang2024@outlook.com (Z.W.); qinxiaotian@bjfu.edu.cn (X.Q.); 2 State Key Laboratory of Efficient Production of Forest Resources, Beijing 100083, China

**Keywords:** root development, functional validation, hormonal regulation, gene expression, transcriptome

## Abstract

Mei (*Prunus mume* Sieb. et Zucc.) is a rare woody species that flowers in winter, yet its large-scale propagation is limited by the variable ability of cuttings to form adventitious roots (ARs). In this study, two cultivars were compared: *P. mume* ‘Xiangxue Gongfen’ (GF), which roots readily, and *P. mume* ‘Zhusha Wanzhaoshui’ (ZS), which is more recalcitrant. Detailed anatomical observations revealed that following cutting, the basal region expanded within 7 days, callus tissues had appeared by 14 days, and AR primordia emerged between 28 and 35 days. Notably, compared to the recalcitrant cultivar ZS, the experimental cultivar GF exhibited significantly enhanced callus tissue formation and AR primordia differentiation. Physiological analyses showed that the initial IAA concentration was highest at day 0, whereas cytokinin (tZR) and gibberellin (GA1) levels peaked at 14 days, with ABA gradually decreasing over time, resulting in increased IAA/tZR and IAA/GA1 ratios during the rooting process. Transcriptomic profiling across these time points identified significant upregulation of key genes (e.g., *PmPIN3*, *PmLOG2*, *PmCKX5*, *PmIAA13*, *PmLAX2*, and *PmGA2OX1*) and transcription factors (*PmWOX4*, *PmSHR*, and *PmNAC071*) in GF compared to ZS. Moreover, correlation analyses revealed that *PmSHR* expression is closely associated with IAA and tZR levels. Overexpression of *PmSHR* in tobacco further validated its role in enhancing lateral root formation. Together, these findings provide comprehensive insights into the temporal, hormonal, and genetic regulation of AR formation in *P. mume*, offering valuable strategies for improving its propagation.

## 1. Introduction

One of the key challenges in ornamental plant breeding and cultivation is developing sustainable methods for growing woody species that can bloom outdoors in relatively low temperatures while maintaining high ornamental value. This is particularly important to meet the global demand for outdoor displays during winter festivities [1]. *Prunus mume* (Sieb. et Zucc.), a deciduous tree in the genus Prunus of the Rosaceae family. It is a renowned ornamental fruit tree prized for its fragrant flowers, vibrant colors, and diverse cultivars. It typically blooms in early spring (January–February), evoking the poetic imagery of “seeking mei in the snow” [2]. Despite being introduced and cultivated for over 3000 years, the propagation techniques for *P. mume* remain incomplete, and the varying cultivation challenges across different cultivars have hindered its broader application [3,4]. Research on cutting propagation in species such as *Prunus armeniaca* and *Prunus cerasus* has demonstrated that Prunus species generally exhibit low rooting success. Rooting efficiency is influenced by multiple factors, including cultivar variations, cutting type, lignification level, exogenous hormone treatments, and environmental conditions [5,6]. This trend is similarly observed in *P. mume* [7]. Current research on *P. mume* propagation predominantly focuses on the effects of plant hormones and cutting substrates on rooting performance. However, there is a lack of in-depth studies examining the underlying mechanisms and factors that contribute to variations in adventitious root (AR) formation.

ARs form from non-root tissues such as stems, leaves, or buds. They can arise spontaneously from stem tissues or be induced by exogenous hormones or mechanical wounding. This process is a complex form of organogenesis co-regulated by both environmental factors and genetic material [8,9]. Anatomical studies have shown that ARs typically originate from cells with regenerative potential, such as those in the vascular cambium or vascular parenchyma [10,11,12]. The development of ARs can be divided into three stages: induction, initiation, and expression [13,14]. Transcriptomic analyses of AR formation in *Petunia hybrida* and *Vigna radiata* have revealed that genes associated with carbohydrate metabolism, amino acid metabolism, lipid metabolism, and protein synthesis are significantly upregulated during the induction phase, whereas genes related to photosynthesis, nitrogen metabolism, and cell wall synthesis are notably downregulated [15,16]. In *Arabidopsis thaliana* leaf explants, the *YUC1* and *YUC4* genes rapidly respond to mechanical wounding caused by leaf excision in both light and dark conditions, promoting auxin synthesis in mesophyll and competent cells. Inhibition of *YUC* gene expression blocks the subsequent expression of *WOX11* [17]. Furthermore, *WOX11* and *WOX12* respond to auxin signals by upregulating *LBD16* and *LBD29*, converting competent cells into root founder cells [18]. In *Populus tomentosa*, overexpression of *PtoWUSa* increases the number of Ars, but shortens their length. The expression of auxin transporter *PIN* genes is downregulated in the ARs of transgenic lines, suggesting that *PtoWUSa* may regulate AR development by modulating polar auxin transport [19].

The formation of adventitious roots (ARs) is regulated by the interplay of multiple hormones. Auxin and cytokinins exhibit antagonistic effects on AR formation [20]. The regulatory mechanism by which auxin and ethylene jointly control AR formation is more intricate. While auxin and ethylene synergistically regulate AR formation in petunia, they exhibit antagonistic effects on adventitious rooting in in vitro *Prunus persica* shoots [11,21]. The endogenous auxin level in plants plays a crucial role in determining the ease with which cuttings can root [22]. Softwood cuttings root more readily than dormant hardwood cuttings, largely due to their higher endogenous auxin levels [23]. High auxin and low cytokinin levels promote AR induction [24]. Mao et al. reported that cytokinins inhibit the formation of apple AR primordia by suppressing the expression of auxin-related genes, thereby reducing endogenous auxin levels [25]. Gibberellins generally promote cell elongation and are considered inhibitors of AR formation. Mauriat et al. demonstrated that overexpression of a key GA biosynthesis gene in hybrid poplar accelerates growth, but reduces rooting efficiency [26]. Abscisic acid (ABA) acts as a negative regulator, inhibiting AR formation [27]. Regarding the mechanism of rooting in *P. mume* cuttings, previous studies have primarily focused on the effects of exogenous hormones, rooting substrates, and cutting times on the propagation of different cultivars [28,29], while molecular-level investigations have been limited.

Cutting propagation is a widely utilized technique for *P. mume* due to its efficiency, cost-effectiveness, and ability to maintain parental genetic traits. However, the molecular and hormonal regulatory networks governing adventitious root (AR) formation remain poorly understood. Comparative analyses of cultivars with differential rooting capacities offer a valuable approach to elucidating the genetic, physiological, and hormonal determinants underlying AR initiation. A deeper understanding of these regulatory mechanisms will facilitate the development of optimized propagation strategies, thereby expanding the horticultural and ecological applications of *P. mume* in urban landscaping and commercial cultivation.

## 2. Results

### 2.1. External Morphology and Anatomical Observation of Adventitious Rooting of Scion

**Figure 1:**
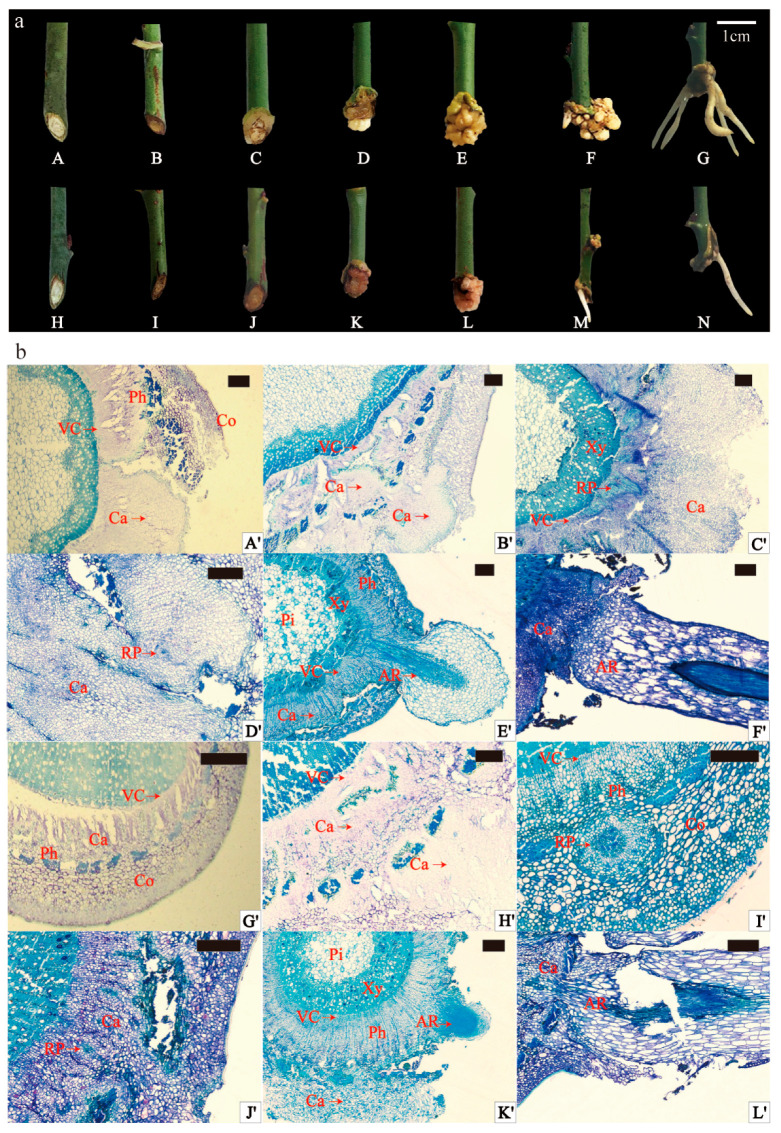
External morphology of GF and ZS scions during rooting, and anatomical changes during the rooting process of semi-tender scions under 12 h photoperiod conditions. (**a**) Figures (**A**–**G**) depict the external morphological changes in GF cuttings, while Figures (**H**–**N**) depict those of ZS cuttings. (**A**,**H**) External morphology before cutting; (**B**,**I**) external morphology 7 days after cutting; (**C**,**J**) external morphology 14 days after cutting; (**D**,**K**) external morphology 28 days after cutting; (**E**,**L**) external morphology 35 days after cutting; (**F**,**M**) external morphology 84 days after cutting; (**G**,**N**) external morphology 120 days after cutting. The scale bar represents 10 mm in (**A**–**N**). (**b**) Anatomical structure during the rooting process of GF and ZS cuttings. Figures (**A**’–**F**’) illustrate the anatomical structure of GD cuttings, while Figures (**G’**–**L’**) depict that of ZS cuttings. (**A**’,**G**’) Anatomical structure 14 days after cutting; (**B**’,**H**’) anatomical structure 28 days after cutting (displaying callus tissue); (**C**’,**I**’) anatomical structure 28 days after cutting (Figure (**C’**) shows root primordia forming at the intersection of the cambium and medullary rays in GF; Figure (**I**) shows root primordia forming in the parenchyma cells of the phloem in ZS); (**D**’,**J**’) anatomical structure 35 days after cutting (root primordia forming in callus tissue); (**E**’,**K**’) anatomical structure 120 days after cutting (Figure (**K’**) shows ARs breaking off); (**F**’,**L**’) ARs originating from callus tissue. The scale bar represents 200 μm (**A**’–**L**’). Abbreviations: AR = adventitious root; Ca = callus; Co = cortex; Ph = phloem; Pi = pith; RP = root primordium; VC = vascular cambium; Xy = xylem.

During rooting under 12 h photoperiod conditions, the external morphological changes in GF and ZS spikes exhibited similarities. Within the initial 7 days, the basal part of the spike underwent expansion. Subsequently, at 14 days, creamy-to-yellowish callus tissues emerged at the spike’s base. Over the next phase (14 days to 35 days), callus tissues proliferated in significant quantities, intensifying in color, and in some instances covering the entire incision. The development of callus tissues gradually slowed between 35 and 84 days. Notably, at this stage, young white ARs began emerging successively from the lower incision of the spike, elongating as they grew (84 to 150 days) (Figure 1a). Comparatively, ZS exhibited less callus tissue formation than GF during the same period post-cutting. Rooting frequency observations revealed that GF exhibited a significantly higher rooting frequency compared to ZS (26.67% ± 4.71% vs. 6.67% ± 2.36%, respectively; *p* < 0.05) (Table S1). Furthermore, observation of stem transverse sections before cutting indicated that both varieties were in the secondary growth stage, with no latent root primordia. In GF, the stem cross section featured a larger proportion of pith, with larger and more loosely arranged pith cells (Figure S1a,d). The cortex, immediately adjacent to the epidermis, consisted of several layers of smaller, more tightly arranged, thicker-walled, angular tissue cells in a ring-like pattern (Figure S1b,e). The pith, centrally located, had a large cell volume, and a circular pith band composed of closely arranged smaller cells connected the pith to the xylem. The pith rays consisted of a single row of cells connecting to the cortex outwardly and the pith inwardly (Figure S1c,f). Both mei varieties demonstrated cortical rooting, with ARs protruding from the lateral side of the stems, and to a lesser extent from callus tissues and the spike’s lower incision. Rooting occurred predominantly in the lateral stem skin, with occasional emergence from callus tissues and the lower spike incision (Figure S2).

Anatomical observations of AR formation in the current-year shoot spike are shown in Figure 1b. In the original cutting stage (OC), both varieties were in the secondary growth stage, characterized by the absence of latent root primordia, indicating an induced rooting type. At 14 days, vascular layer cells in the cuttings began dividing, forming callus tissues (Figure 1b(A’,G’)). At day 28, callus tissues were continuing to develop. Parenchyma cells in the phloem and cortex underwent division, contributing to callus formation stage (CF) at the spike’s cutting site (Figure 1b(B’,H’)). In GF, a distinct cluster of smaller, darker-stained cells appeared at the junction between the cambium layer and pith rays, marking the initiation of the root primordium formation stage (RP) (Figure 1b(C’)). In ZS, AR primordia developed more rapidly, forming rounded structures in the phloem’s thin-walled cells (Figure 1b(I’)). By day 35, small, less differentiated, subrounded root primordia were evident in the callus tissues of both varieties (Figure 1b(D’,J’)). At 120 days, ARs emerged through the epidermis in both varieties (Figure 1b(E’,K’)). In GF cuttings, root primordia originated from two distinct sites: the callus tissues and the cambium–pith ray junction. These primordia subsequently developed into ARs protruding from the stem surface (Figure 1b(E’,F’)). In ZS, root primordia primarily arose from phloem parenchyma and callus tissues, with subsequent elongation into ARs (Figure 1b(K’,L’)). The results highlighted that ARs originated from three primary sites: thin-walled parenchyma cells at the cambium–medullary ray junction, phloem parenchyma cells, and callus tissues. Notably, spikes at the base typically produced more and longer roots, with root elongation more pronounced in areas with less callus tissue.

### 2.2. Transcriptome Sequencing Overview and Differential Expression Analysis

**Figure 2:**
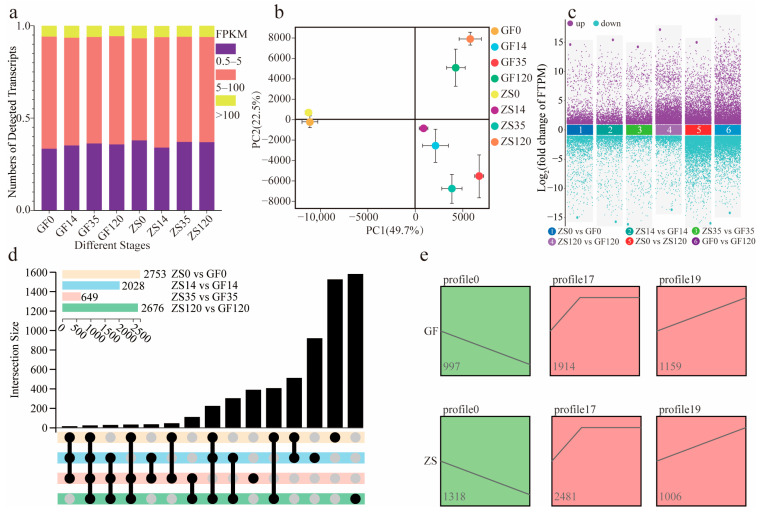
Preliminary transcriptomic analysis of GF and ZS. (**a**) Transcript abundance in each sample. (**b**) Principal component analysis of RNA-seq data. The PC1 and PC2 coordinates represent the first and second principal components, respectively. Colored points correspond to distinct samples, as indicated in the legend. (**c**) Scatterplot illustrating differential expression across multiple groups. The vertical axis represents the logarithmic value of fold change (log_2_FC), and the horizontal axis shows the names of comparison groups. (**d**) Venn diagram illustrating differentially expressed transcripts across the four flowering stages in GF and ZS varieties. (**e**) Trend clustering of differentially expressed genes during AR formation in two *P. mume* cultivars.

The sequencing data statistics are shown in Table S2. A total of 44.18–67.22 million clean reads were obtained, with 96.99–98.11% and 91.53–94.07% of the clean bases meeting the Q20 and Q30 quality thresholds, respectively, indicating high sequencing quality (Table S2). The overall alignment rate of clean reads to the *Prunus mume* reference genome ranged from 83.18% to 94.34% (Table S3). Approximately 35.11% of the expressed genes exhibited expression levels between 0.5 and 5.0 FPKM, while 58.25% had expression levels between 5.0 and 100.0 FPKM (Figure 2a). Principal component analysis (PCA) results are presented in Figure 2b. The first principal component (PC1) explained 49.70% of the variance, whereas the second principal component (PC2) explained 22.50%. The positions of ZS0 and GF0 differed substantially from those of ZS14 and GF14. Similarly, ZS35 and GF35 exhibited significant divergence compared with ZS120 and GF120. This indicates distinct transcriptional differences between the CF and OC, as well as between the AR formation stage (AF) and RP.

During AF, the number of differentially expressed genes (DEGs) between cultivars at the same time point was smaller than that between the OC and AF within each cultivar (Figure 2c). The OC and AF exhibited the highest numbers of DEGs, with 2753 and 2676 genes, respectively. Twenty genes were differentially expressed across all four stage comparisons. Additionally, 508 genes were differentially expressed between the OC and CF, which corresponds to the first two stages of AR formation (Figure 2d). A trend analysis of all DEGs involved in AF grouped the genes into 20 distinct profiles (Figure S3). Among these, profiles 0, 17, and 19 were significantly enriched, warranting further investigation due to their potential roles in root development. In ZS and GF, 1372 and 1035 genes, respectively, displayed a persistent downregulation trend in profile 0. In profile 17, 2843 and 2088 genes were upregulated during the CF and subsequently maintained high expression levels. In profile 19, 1041 and 1225 genes were continuously upregulated, potentially indicating involvement in later stages of root formation (Figure 2e).

### 2.3. Annotation and Classification of Differentially Expressed Genes

**Figure 3:**
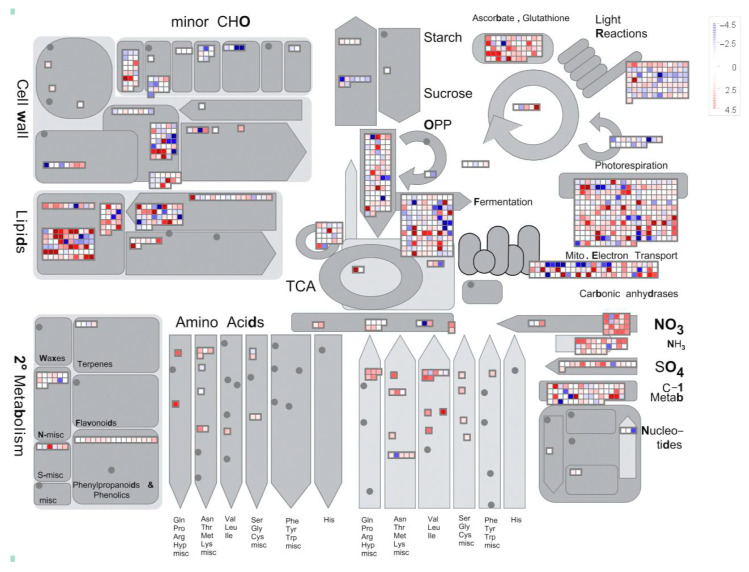
MapMan analysis of candidate differentially expressed genes. The metabolic overview depicts candidate genes categorized into 16 processes: cell wall metabolism, small carbohydrate metabolism, starch metabolism, sugar metabolism, photosynthesis including light reactions, tetrapyrroles, Calvin cycle and photorespiration, glycolysis, metabolism, citric acid cycle, oxidative pentose phosphate pathway, mitochondrial electron transport, amino acid biosynthesis, nucleotide metabolism, lipid metabolism, and secondary metabolism.

Among the 7587 differentially expressed genes (DEGs), 7162 were successfully annotated in the Gene Ontology (GO) database based on sequence homology. The entire *P. mume* genome was uploaded to the PlaBi Database (Plant Database) for online annotation (https://www.plabipd.de/portal/mercator-sequence-annotation/, accessed on 25 April 2024), and a comprehensive metabolic overview was generated using MapMan (Figure 3). As shown in the metabolic map, most DEGs were associated with secondary metabolism, lipid metabolism, cell wall metabolism, and photosynthetic processes. During AF in *P. mume*, cells appeared to complete cell division, with only 122 DEGs remaining active in cell wall development pathways. When mapped to the “Regulation Overview” (Figure S4), 69 DEGs were related to hormone biosynthesis, including 11 involved in auxin (IAA) biosynthesis, 35 in abscisic acid (ABA), 2 in cytokinin (CK), and 16 in gibberellin (GA) metabolism. Notably, the enrichment of IAA-related DEGs suggests a potential role for auxin in coordinating cell differentiation during root development. These GO annotations collectively provide insights into the molecular mechanisms underlying AR formation in *P. mume*.

Additionally, 5343 genes from the two *P. mume* cultivars were mapped to 132 KEGG metabolic pathways (Table S4). The significantly enriched pathways included the “citrate cycle (TCA cycle)” (ko00020) and “proteasome” (ko03050), along with pathways involved in the “biosynthesis of secondary metabolites” (ko01110) and “metabolic pathways” (ko01100). Genes exhibiting continuous upregulation were significantly enriched in pathways such as “phenylpropanoid biosynthesis” (ko00940), “carbon metabolism” (ko01200), and “tyrosine metabolism” (ko00350), suggesting that these metabolic processes may play key roles in AR formation (Figure S5).

To further elucidate the candidate genes involved in AR formation in semi-softwood cuttings of *P. mume,* DEGs associated with key components of plant hormone signaling pathways were analyzed. In total, 26, 23, 14, and 13 DEGs were identified in the auxin, cytokinin (CK), gibberellin (GA), and abscisic acid (ABA) signaling pathways, respectively. The expression trends of these genes were generally consistent between GF and ZS; however, certain genes, such as *AUX/IAA, SAUR*, *GH3*, *LOG*, *AHK*, *SCL*, and *PYR/PYL*, exhibited higher expression levels in GF than in ZS. Similarly, genes known to regulate AR formation, including *PmSHR*, *PmPIN*, and *PmNAC*, also showed higher expression levels in GF (Figure S6). This consistent upregulation of hormone-related DEGs suggests potential differences in hormonal sensitivity and signaling efficiency between the two cultivars, which may underlie their distinct rooting capacities.

### 2.4. Identification of DEGs Related to Root Developmental Regulation Across Different Stages

**Figure 4:**
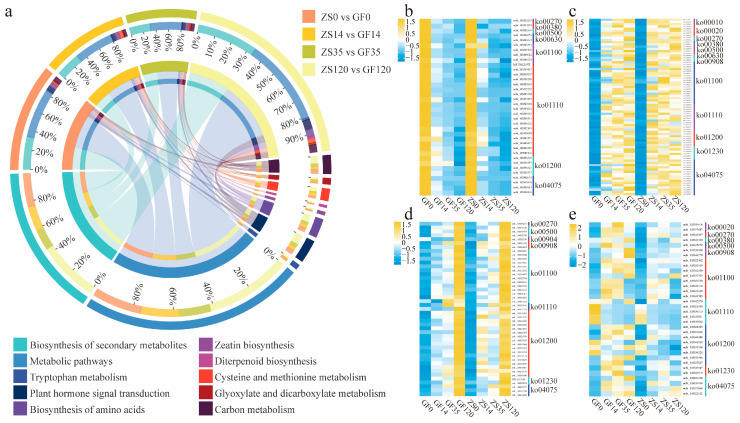
Comparative heatmaps of differentially expressed genes enriched in key pathways of AR formation at different rooting stages. (**a**) Enrichment of differentially expressed genes in pathways related to AR formation; (**b**) genes consistently downregulated and enriched in key pathways; (**c**) genes initially upregulated and then consistently expressed, enriched in key pathways; (**d**) genes continuously upregulated and enriched in key pathways; (**e**) genes with higher expression levels in GF than in ZS across all four stages. The corresponding gene IDs can be found in Table S6.

During the four rooting stages—OC, CF, RP, AF—in GF and ZS, we identified 509, 415, 335, and 954 differentially expressed genes (DEGs), respectively. These DEGs were significantly enriched in 13 distinct metabolic pathways (Figure 4a). Among the pathways with a high number of DEGs associated with AR formation were “Biosynthesis of secondary metabolites”, “Metabolic pathways”, “Plant hormone signal transduction”, and “Starch and sucrose metabolism”. To further investigate the roles of these genes, we performed a logical intersection (Boolean operation) between DEGs enriched in these pathways and those exhibiting significant expression changes during AF. As a result, 173 genes were identified as involved in phytohormone biosynthesis, metabolism, and signal transduction. These included 20 genes associated with ethylene, 50 with auxin, 26 with abscisic acid (ABA), 36 with brassinosteroids, 14 with gibberellins (GA), and 27 with cytokinins (CTK) (Table S5). Analysis of these genes revealed distinct expression patterns related to AR formation. Specifically, 36 genes in these pathways exhibited continuous downregulation (Figure 4b), suggesting their potential roles in repressing inhibitory factors of root formation. Additionally, 79 genes were initially upregulated and subsequently maintained high expression levels (Figure 4c), indicating their likely involvement in the early induction phase of ARs. In contrast, 45 genes displayed sustained upregulation throughout the process (Figure 4d), highlighting their potential roles in supporting cell differentiation and root elongation. Notably, we identified 34 DEGs that consistently showed higher expression in GF compared to ZS across all four stages of AR formation (Figure 4e). These genes are likely critical in elucidating the molecular basis of the observed differences in rooting capacity between these two *P. mume* cultivars.

### 2.5. Co-Expression Analysis and Validation of Key Hormone Levels and Related Genes

**Figure 5:**
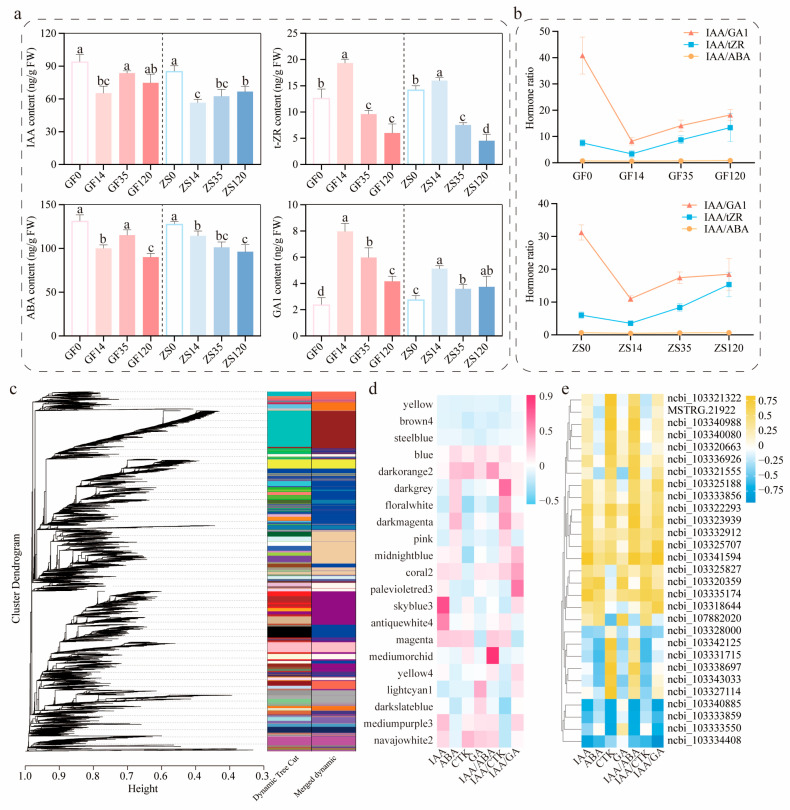
Dynamic changes in phytohormone concentrations and correlation analysis during AR formation in semi-hardwood cuttings of *P. mume*. (**a**) Concentration dynamics of auxin (IAA), cytokinin (tZR), abscisic acid (ABA), and gibberellin (GA1). (**b**) Dynamics in the ratios of auxin (IAA) to other phytohormones (tZR, GA1, and ABA represent cytokinin, gibberellin, and abscisic acid, respectively). (**c**) Hierarchical clustering tree from the weighted gene co-expression network analysis. Each color represents a corresponding gene expression module. (**d**) Correlation analysis between expression modules and key hormones. (**e**) Correlation analysis between important candidate genes and hormones. Error bars indicate SE (*n* = 3). Different letters denote significant differences at *p* < 0.05 analyzed by Tukey’s test.

The concentrations of plant hormones measured in cuttings at different stages varied significantly (Figure 5a, *p* < 0.05). Auxin concentration, indicated by IAA levels, peaked at the OC. During the process of AR formation, cytokinin (CTK; measured as tZR) and gibberellin (GA1) concentrations exhibited similar dynamic changes, both reaching their highest levels on day 14. Notably, the concentration of abscisic acid (ABA) remained significantly higher than that of other plant hormones during AR formation, gradually declining as the process progressed. The ratios of IAA/tZR and IAA/GA1 showed a continuous upward trend throughout AR formation. By day 120, compared with day 14, the IAA/tZR ratio had increased 3.96-fold and 4.33-fold in GF and ZS, respectively. Similarly, the IAA/GA1 ratio increased by 2.21-fold and 1.68-fold in GF and ZS, respectively (Figure 5b). These hormonal shifts suggest an increasing dominance of auxin-mediated signaling during later stages of AR formation.

To comprehensively understand the gene expression patterns during AR formation and identify genes associated with this process, we performed weighted gene co-expression network analysis (WGCNA) with a power parameter of 8 (Figure 5c). This analysis identified 21 functional gene modules, which were subsequently correlated with hormone level dynamics. The results showed that changes in ABA levels were significantly associated with the skyblue3 module, while ABA and CTK levels correlated with the darkorange2 and blue modules. GA levels were most closely associated with the lightcyan1 module (Figure 5d). Through this analysis, 28 key candidate genes were identified, including 20 structural genes and 8 transcription factors (Table S7).

Correlation analysis between the expression levels of these candidate genes and hormone changes revealed several significant associations (*p* < 0.05). The genes *PmLAX3*, *PmPIN3*, *PmSHR*, and *PmIAA14* exhibited significant positive correlations with IAA level changes. Additionally, *PmNAC029* showed positive correlations with IAA and tZR levels, but negative correlations with ABA and GA levels. Similar correlation patterns were observed for gibberellin, with *PmGA20X1* as the key structural gene and *PmSLC14*, *PmSLC6*, and *PmSLC7* as associated transcription factors. For cytokinin, *PmCKX7* was identified as the key structural gene, with *PmNAC071* as the corresponding transcription factor. Regarding late-stage auxin signaling, the key genes *PmIAA13* and *PmIAA6*, along with the transcription factor *PmNAC071*, exhibited significant associations with IAA dynamics. The balance of endogenous hormones, particularly the relative levels of auxin, cytokinin, gibberellin, and ABA, was identified as a critical factor influencing AR initiation and development. Specifically, *PmPIN3*, *PmIAA14*, and *PmSHR* were significantly associated with the balance between IAA and indole-3-butyric acid (IBA). In addition, *PmGA2OX1* and *PmGH3*.1 exhibited significant correlations with changes in the IAA/tZR ratio, while *PmLOG2*, *PmPIN3*, and *PmIAA16* were closely associated with changes in the IAA/GA1 ratio. Collectively, these findings support the hypothesis that these genes play regulatory roles in the AR formation process of *P. mume,* potentially through hormone-mediated signaling pathways (Figure 5e).

### 2.6. Construction of Gene Co-Expression Networks

**Figure 6:**
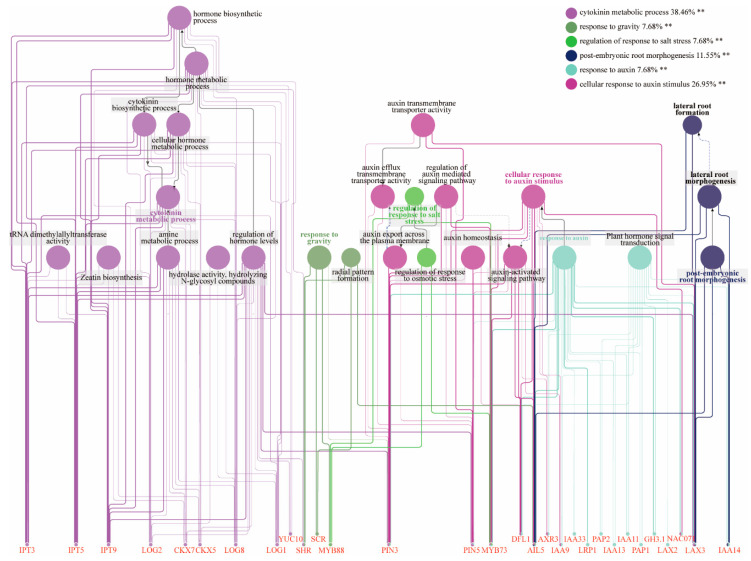
Interaction network analysis of key genes. Key genes were subjected to analysis using Cytoscape 3.8.2, revealing significant enrichment across 30 functional categories. ** Highly significant enrichment—*p* < 0.01.

To further investigate the potential roles of the key transcription factors and structural genes identified, an interaction network was constructed using Cytoscape software (Figure 6). The analysis revealed significant gene enrichment in six major functional categories. Among these, 38.46% of the genes were involved in the cytokinin metabolic process, while 26.95% participated in cellular responses to auxin stimuli. Additionally, 11.55% of the genes were associated with post-embryonic root morphogenesis, whereas 7.68% were related to each of the following processes: response to gravity, regulation of response to salt stress, and response to auxin. The results indicated that *IPT3*, *IPT5*, *IPT9*, *LOG2*, *CKX7*, *CKX5*, *LOG8*, *LOG1*, and *YUC10* were significantly enriched in cytokinin metabolic processes, hormone level regulation, and cellular hormone metabolism. These genes collectively play a crucial role in cytokinin biosynthesis, likely by modulating cytokinin interconversion and degradation to maintain hormone homeostasis during AR development in *P. mume*. In addition, the genes *SHR*, *SCR*, and *MYB88* were enriched in processes related to gravity sensing and radial pattern formation. This enrichment suggests that these genes may regulate the spatial organization of root tissues and contribute to the morphogenesis of ARs in *P. mume*. More importantly, *AIL5* and *LAX3* were concurrently enriched in radial pattern formation, cellular response to auxin stimulus, response to auxin, and lateral root formation. This concurrent enrichment indicates that these genes may serve as key regulators in the spatial patterning and formation of ARs. Furthermore, genes such as *IAAs*, *LAX2*, *GH3*.1, and *NAC071* were enriched in processes related to auxin signaling and plant hormone transduction. This enrichment suggests that these genes may regulate auxin homeostasis and mediate hormonal cross talk, potentially by modulating gene expression patterns involved in root primordium and AR formation.

### 2.7. Overexpression of PmSHR Increases the Number of Lateral Roots in Tobacco

**Figure 7:**
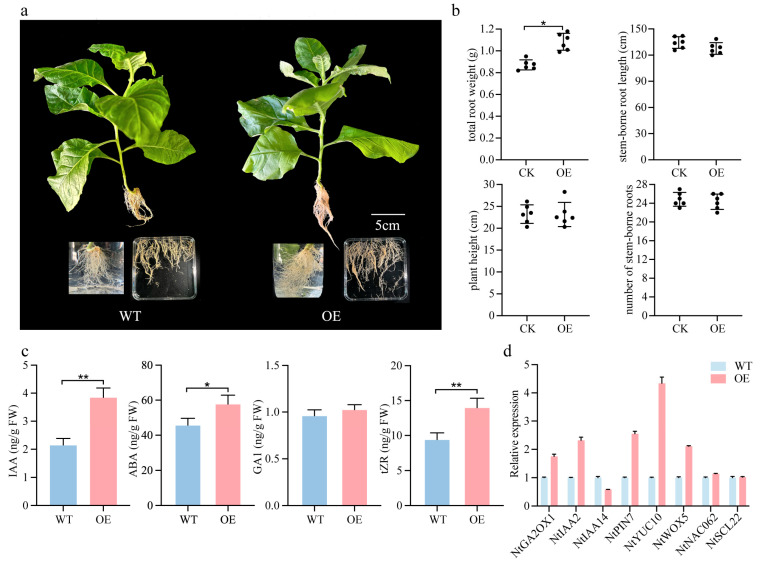
Phenotypic characterization of tobacco plants overexpressing *PmSHR*. (**a**) Wild-type (WT) and OE-*PmSHR* tobacco plants; (**b**) key phenotypic differences between WT and OE-*PmSHR* lines; (**c**) content of IAA, ABA, GA, and tZR in WT and OE-*PmSHR* plants; (**d**) expression levels of genes related to AR formation in the roots of WT and transgenic tobacco lines. Asterisks indicate statistical significance between the two datasets: *p* < 0.05 (*), *p* < 0.01 (**).

Compared with the control group, OE-*PmSHR* plants did not exhibit significant differences in root length, plant height, or the number of roots formed along the stem. However, these transgenic plants displayed significantly greater root biomass, suggesting that *PmSHR* overexpression promotes lateral root formation in tobacco (Figure 7a and Figure S7). In addition, the levels of indole-3-acetic acid (IAA), abscisic acid (ABA), and trans-zeatin riboside (tZR) were all significantly higher in the OE-*PmSHR* lines than in the wild type (Figure 7b). RT-PCR analyses revealed that *NtGA2OX1*, *NtIAA2*, *NtPIN7*, *NtYUC10*, and *NtWOX* were significantly upregulated. In contrast, the expression of *NtIAA14* was downregulated, which may relieve auxin-mediated growth inhibition. The expression of *NtNAC062* and *NtSCL22* remained unchanged (Figure 7c,d). These findings suggest that the heterologous expression of *PmSHR* contributes to lateral root development, potentially by modulating auxin signaling and lateral root primordium formation. However, the rooting process is likely regulated by a complex genetic network involving multiple hormone pathways. Further investigations into the interactions between *PmSHR* and downstream genes, such as *NtIAA14* and *NtPIN7*, may provide more insights into the molecular mechanisms underlying root development.

## 3. Discussion

### 3.1. Anatomical Structures and Rooting Patterns in Adventitious Root Formation of P. mume Cuttings

In studies examining the factors influencing adventitious root (AR) formation in cuttings, plant anatomists have proposed that the anatomical structure of woody plant stems plays a critical role in rooting ability. Specifically, the presence of multiple layers of sclerenchyma tissue in the stem cortex or a continuous ring of sclerenchyma impedes AR formation. Conversely, stems that lack such extensive sclerenchyma are more conducive to rooting [30,31]. Examination of the first-year stems of two *P. mume* cultivars, GF and ZS, revealed that by late August, these stems were undergoing secondary growth, and no latent root primordia were observed in the cuttings. This finding contrasts with genera such as *Populus* [32] and *Juniperus* [33], which develop latent root primordia early in stem development. In contrast, species like *Carpinus betulus* [34], *Platycladus orientalis* [35], *Robinia pseudoacacia* L. [36], and *Acer rubrum* [37] form induced root primordia during the cutting process itself. In Ribes nigrum, the type of root primordium formed depends on the degree of stem maturation: latent root primordia are present in woody, mature first-year stems, but absent in younger stems [38].

Generally, cutting types are classified into phloem-derived rooting, callus-derived rooting, or mixed rooting. Dong [39] observed that cuttings from two *P. mume* cultivars, *meiren* and *sanlun yudie*, belong to the callus-derived rooting type. In contrast, both GF and ZS exhibited a mixed rooting pattern, with AR primordia originating from multiple sites. Specifically, in GF, root primordia formed at the junction of the cambium and medullary rays as well as within callus tissue. In ZS, primordia originated either from phloem parenchyma cells or from callus tissue. Therefore, for late-August cuttings of P. mume, AR formation occurs at multiple sites, arising from parenchymal cells at the cambium–medullary ray junction, phloem parenchyma cells, and callus tissue. In this study, the rooting process in GF and ZS cuttings was divided into three stages: cutting formation (CF), root primordium (RP) formation, and adventitious root (AF) development. The AF stage (35–120 days) was relatively prolonged, indicating a slower rooting process when cuttings were taken in late August. Under a 12 h photoperiod, root primordia had formed by mid-October; however, the significant drop in temperature likely reduced cellular division activity, thereby slowing root primordium cell proliferation and extending the overall rooting period [40].

### 3.2. Endogenous Hormonal Regulation and Gene Expression in Adventitious Root Formation of P. mume

Endogenous hormones play a pivotal role in adventitious root (AR) formation by regulating processes such as root primordium induction, callus formation, root elongation, and root morphology [41,42]. Abscisic acid (ABA) is a key regulator of root formation, primarily by inhibiting root growth and maintaining bud dormancy [43]. In this study, ABA levels progressively decreased during rooting. Research on *Arabidopsis thaliana* has shown that high ABA levels suppress the polar transport and signaling of indole-3-acetic acid (IAA), thus maintaining cellular dormancy while potentially creating conditions conducive to root primordium differentiation [44]. As rooting progresses, the concurrent decline in both ABA and IAA may help maintain a favorable IAA/ABA ratio for AR development. The reduction in ABA alleviates its inhibition of the IAA signaling pathway, promoting IAA accumulation and polar transport within the root primordium, thereby facilitating root elongation [22]. In this study, gibberellin (GA_1_) concentrations in both GF and ZS cultivars showed an initial increase followed by a subsequent decrease, suggesting its involvement in early root initiation, but potential inhibition during later stages. The role of gibberellins (GAs) in AR formation in woody plants remains controversial. In *Solanum tuberosum*, elevated GA_3_ levels promote callus formation in tubers, facilitating AR development [45]. In contrast, in *Phyllanthus amarus* and *Camellia sinensis*, GA_3_ inhibits root formation by suppressing primordium cell division and interfering with IAA-induced root initiation [46,47].

In auxin biosynthesis, YUC catalyzes the conversion of indole-3-pyruvate (IPA) into IAA [17,48]. During AR formation in *P. mume* cuttings, *PmYUC10* reached its highest expression level in the root primordium (RP) of the GF cultivar, suggesting that competent cells may be synthesizing auxin at this stage. Auxin polar transport relies on influx carriers from the *AUX/LAX* family, efflux carriers from the *PIN* family, and the ABCD/MDR/PGP family, which mediate both influx and efflux [49]. In this study, *PmLAX2* and *PmPIN1A* were continuously upregulated in both *P. mume* cultivars, with higher expression levels in GF compared to ZS. Additional genes encoding auxin response proteins (e.g., AUX/IAA, GH3, ARG, SAUR) also exhibited varying degrees of expression during the adventitious root (AF) stage. These findings suggest that these genes contribute to AR formation in *P. mume* and may be critical factors underlying the differing rooting capacities of the two cultivars. In the cytokinin biosynthesis pathway, *IPT* genes encode rate-limiting enzymes, while *LOG* genes convert cytokinins to their active, free forms [50]. In this study, *PmIPT3* was significantly upregulated during the cutting formation (CF) stage in ZS, whereas *PmLOG1* and *PmLOG3* showed relatively high expression levels in GF at the same stage, suggesting that cytokinins may regulate callus meristem activity [51]. Cytokinin oxidase (CKX) genes, which encode cytokinin-degrading enzymes, were highly expressed during the AF stage in both cultivars, with *PmCKX5* and *PmCKX7* being particularly prominent. This suggests a low accumulation of cytokinins at this stage, potentially influencing root elongation.

Collinearity analysis of the key candidate genes revealed that genes related to root formation exhibit more extensive homologous relationships in dicotyledons compared to monocotyledons (Figure S8). Furthermore, during the evolutionary transition from woody to herbaceous plants, genes associated with root formation may have undergone both contraction and expansion [52].

### 3.3. Hormonal Ratios and Gene Interactions in the Regulation of Adventitious Root Formation in P. mume

Studies have shown that the formation of adventitious roots (ARs) is regulated by the complex interactions and balance among various hormones, with auxin serving as a central regulator in this process [36]. Research indicates that the ratios of auxin to other hormones are closely linked to the potential for AR formation [53]. In this study, the IAA/tZR and IAA/ABA ratios were higher in the GF compared to ZS. Previous studies have demonstrated that higher ratios of auxin to cytokinins, as well as higher ratios of auxin to abscisic acid (ABA), are associated with better rooting rates [36,54]. During the AR formation process in semi-lignified cuttings of the *P. mume* cultivars ZS and GF, the IAA/tZR ratio initially decreased and then increased. This suggests that a lower IAA/tZR ratio may be more favorable for root primordium formation in *P. mume* cuttings, while a higher IAA/tZR ratio promotes callus formation and root elongation. Additionally, the trend in the IAA/GA ratio mirrored that of the IAA/tZR ratio, being higher in GF than in ZS. This difference may explain why GF is more prone to forming ARs than ZS. Similar results have been reported in studies on rooting rates in *C. sinensis* cuttings [55] and *Malus pumila* tissue culture seedlings [56]. Regarding the interaction between IAA and ABA, recent studies have shown that PYLs can interact with MYBs to enhance the expression of ARFs and work together with downstream LBDs in the positive regulation of auxin biosynthesis during root growth [57]. Additionally, *SAUR19* has been implicated in root development in *A.* thaliana [58]. Based on these findings, we hypothesize that PYLs, along with downstream *SAURs* and *ARF/IAA* complexes, may mediate the signal interactions between auxin and ABA. In this study, we confirmed that *PmPYLs*, *PmARFs*, *PmIAAs*, and *PmSAURs* are strongly correlated with the interactions between auxin and ABA during AR formation in semi-lignified *P. mume* cuttings.

### 3.4. Role of the SHR Gene in Adventitious Root Formation

The mechanisms by which the *SHR* influences root growth and development in plants are currently under extensive investigation; however, its role during the rooting process of cuttings remains unexplored [59,60,61]. This study demonstrates that the transcription factor encoded by the *SHR* plays a critical role in cell division and tissue expansion in roots during the adventitious rooting of *P. mume* cuttings. Modulating the expression of *SHR* significantly impacts the formation of lateral roots. *SHR* is expressed in the central vascular tissue of roots, and its protein product moves to the cortex/endodermis initial (CEI) cells, where it activates the *SCR*, thereby regulating the division of CEI cells and the development of cortical and endodermal cell layers [62,63]. The critical regulatory role of *SHR* in cortical cell division has been validated in *A. thaliana*, *Oryza sativa*, and other plants [64]. It can be hypothesized that during the cutting process of *P. mume, SHR* functions as a radial signal gene, regulating the establishment of radial patterns in the plant and consequently influencing the formation of lateral roots. Furthermore, the functionality of *SHR* during adventitious rooting in *P. mume* likely depends on its interactions with hormones, other functional genes, and transcription factors, thereby forming a complex regulatory network. Therefore, overexpressing a single gene may not significantly alter the plant’s complex traits, and the overall effects of gene networks and signaling pathways must be considered [65].

## 4. Materials and Methods

### 4.1. Experimental Materials

Two *P. mume* cultivars with distinct rooting efficiencies, GF (*P. mume* ‘Xiangxue Gongfen’, easy to root) and ZS (*P. mume* ‘Zhusha Wanzhaoshui’, recalcitrant) (Table S1), were selected using semi-lignified first-year shoots. Morphological and anatomical observations were conducted at 7-day intervals until AR formation. For molecular analyses, samples were collected at four time points post-cutting: day 0 (original cutting, OC), day 14 (callus formation stage, CF), day 35 (root primordium stage, RP), and day 120 (adventitious root formation stage, AF), labeled GF0, GF14, GF35, GF120 and ZS0, ZS14, ZS35, ZS120. During sampling, the basal 1 cm of each cutting (excluding the xylem) was excised, washed with distilled water, quickly frozen in liquid nitrogen, and stored at −80 °C. Each time point included three biological replicates (five cuttings per replicate).

### 4.2. Cutting Conditions

Healthy, disease-free semi-lignified first-year shoot tips were uniformly trimmed to 10 cm with a diagonal lower cut and two functional leaves retained. Prior to cutting, seedling pots, substrates, and surrounding areas were sterilized with an 800-fold carbendazim solution. The cuttings were then immersed in the same solution for 15 min, surface-dried, and subsequently dipped in a 2000 mg·L^−1^ IBA solution for 8 s before being inserted into a mixed substrate of peat and perlite (2:1, *v*/*v*) at a depth of 3–4 cm. The substrate pH was adjusted to 6.0–6.5. Following insertion, an LK-100 full-light mist sprayer was used to maintain controlled environmental conditions, with the temperature set to 25 ± 2 °C for the first 60 days, followed by 15 ± 2 °C until the end of the experiment in response to seasonal temperature decline, light intensity at approximately 200 μmol·m^−2^·s^−1^, a photoperiod of 12 h light/12 h dark, and relative humidity maintained between 75% and 90%. Spraying was conducted every 15 min, with durations adjusted from 10 s during the first 15 days, to 6 s for days 15–30, and finally 3 s after day 30.

### 4.3. Morphological and Anatomical Analysis of Adventitious Root Formation in P. mume Cuttings

For sampling, a 1 cm stem segment from the base of each cutting was excised and immersed in FAA fixation solution (70% ethanol–formalin–acetic acid = 90:5:5, *v*/*v*/*v*) for 48 h. Hard tissue sections were prepared by dehydrating and clearing the fixed stem segments, followed by infiltration with plastic resin and embedding in methyl methacrylate (MMA). Sections were then cut using a Leica microtome, de-embedded with ethylene glycol diethyl ether acetate, rehydrated, stained with toluidine blue, cleared with a clarifying agent, and mounted with coverslips. The prepared sections were observed and photographed under a microscope to analyze the morphological and anatomical aspects of AR formation.

### 4.4. Quantitative Analysis of Endogenous Hormones

Based on the method of Ljung et al. [66] with modifications, gibberellin GA_1_ (a type of GA), indole-3-acetic acid (IAA), trans-zeatin riboside (tZR, a type of cytokinin CK), and abscisic acid (ABA) were extracted. The quantification of these hormones was performed via high-performance liquid chromatography (HPLC) following the protocol of Van et al. [67]. The purification was carried out using a Poroshell 120 SB-C18 column, and the analysis was conducted using liquid chromatography–tandem mass spectrometry (LC-MS, Agilent 1290-6460A, Agilent Technologies, Palo Alto, CA, USA) in conjunction with the SCIEX 6500Qtrap system (Framingham, MA, USA).

### 4.5. RNA Isolation, Transcriptome Sequencing, and Differential Gene Expression Analysis

Transcriptome library construction and Illumina sequencing were performed by Guangzhou Jidiao Biotechnology Co., Ltd. (Guangdong, China). Effective reads from the samples were aligned to the *P. mume* reference genome (BioProject = PRJNA171605) using HISAT2 [68]. Based on the HISAT2 alignment results, transcriptomes were reconstructed using StringTie, and the expression levels of all genes in each sample were calculated [63]. Gene expression abundance was measured using the FPKM (fragments per kilobase of transcript per million mapped reads) method [69].

Differential gene expression analysis was conducted using DESeq2 (version 1.46.0), with criteria set at a false-discovery rate (FDR) < 0.05 and |log_2_ fold change (FC)| > 1. Differentially expressed genes (DEGs) were mapped to entries in the Gene Ontology (GO) database (http://www.geneontology.org/, accessed on 5 March 2024) and annotated. Enrichment analysis of DEGs was performed against the KEGG database (https://www.kegg.jp/kegg/, accessed on 7 March 2024). Weighted gene co-expression network analysis (WGCNA) was performed as per Langfelder and Horvath [70]. The interaction network was analyzed using Cytoscape 3.8.2. Metabolic overview maps of DEGs were constructed using MapMan 3.7.0 [71].

### 4.6. Tobacco Transformation

Following the methodology of Anwar et al. [72], the 35S::GFP-PmSHR vector was introduced into tobacco (*Nicotiana tabacum*) through Agrobacterium-mediated transformation. In the T_0_ generation, genomic DNA was screened using PCR to identify positive transgenic plants. The T_1_ generation transgenic lines were subsequently cultured under long-day conditions (16 h light/8 h dark, 25 °C/18 °C) for six weeks. After this cultivation period, phenotypic data were collected and quantitative real-time fluorescence PCR (qRT-PCR) analyses were conducted on the obtained plants.

### 4.7. Quantitative Real-Time PCR Validation

First-strand cDNA synthesis for all samples was performed using PrimeScript RT Master Mix (TaKaRa, Kusatsu, Japan, https://www.takarabio.com/, accessed on 6 August 2024). Expression levels were quantified on a Bio-Rad CFX96 real-time PCR machine (Bio-Rad, Hercules, CA, USA, https://www.bio-rad.com/, accessed on 8 August 2024) utilizing SYBR Fast qPCR Mix (TaKaRa). Relative expression levels were calculated using the 2^−ΔΔCt^ method, with the tea plant *β-actin* gene (CsActin; accession HQ420251) serving as the internal control. The RT-qPCR protocol included an initial denaturation step at 95 °C for 5 min, followed by 40 cycles of denaturation at 95 °C for 10 s, annealing at 58 °C for 20 s, and extension at 72 °C for 20 s. Specific primers were designed using PRIMER 5 (Table S8) and synthesized by Sangon Biotech, Shanghai, China (https://www.sangon.com). All reactions were performed in triplicate to ensure accuracy and reproducibility.

## 5. Conclusions

This study elucidates the anatomical and molecular mechanisms governing AR formation in two *P. mume* cultivars, GF and ZS, with distinct rooting efficiencies. Anatomical analysis identified a mixed rooting pattern originating from cambium-medullary ray intersections, phloem parenchyma cells, and callus tissue. Transcriptomic profiling revealed key differentially expressed genes involved in auxin, cytokinin, and gibberellin pathways, with GF exhibiting higher IAA/tZR and IAA/ABA ratios, correlating with its significantly higher rooting frequency compared to ZS, indicating enhanced rooting ability. WGCNA pinpointed 28 candidate genes, including *PmYUC10*, *PmPIN1A*, and *PmSHR*, essential for AR formation. Functional validation through *PmSHR* overexpression in tobacco increased lateral root numbers, highlighting its regulatory role. These findings provide a foundation for optimizing *P. mume* propagation. Future research will focus on applying these insights to develop efficient propagation protocols, enhancing cultivation practices and promoting broader agroecosystem benefits. 

## Data Availability

The datasets generated and/or analyzed during this study are available from the CNCB repository (BioProject ID: CRA013634) (https://www.cncb.ac.cn/, accessed on 27 November 2023). Public access to the databases mentioned above are open, and no administrative permissions are needed for accessing and using the data. Material samples are available from authors.

## Supplementary Materials

The following supporting information can be downloaded at https://www.mdpi.com/article/10.3390/ijms26062416/s1.

## Abbreviations

The following abbreviations are used in this manuscript:

IAA	indole-3-acetic acid
PIN	puroindoline
LOG	cytokinin-activating enzyme; Lonely Guy
CKX	cytokinin oxidase
LAX	lax panicle
GA2OX	GA2-oxidase
WOX	WUSCHEL-related homeobox
SHR	SHORT-ROOT
NAC	no apical meristem
SCL	SCARECROW-like
ABA	abscisic acid
tZR	trans-zeatin nucleoside
YUC	YUCCA
