# Supplementary material for: Investigating the Mechanisms of Adventitious Root Formation in Semi-Tender Cuttings of Prunus mume: Phenotypic, Phytohormone, and Transcriptomic Insights
Source: Int J Mol Sci. 2025 Mar 7;26(6):2416. doi: 10.3390/ijms26062416 (PMC11941866; doi:10.3390/ijms26062416)
Supplement: Supplementary file 1 [file ijms-26-02416-s001.zip › Supplementary figures.pdf]

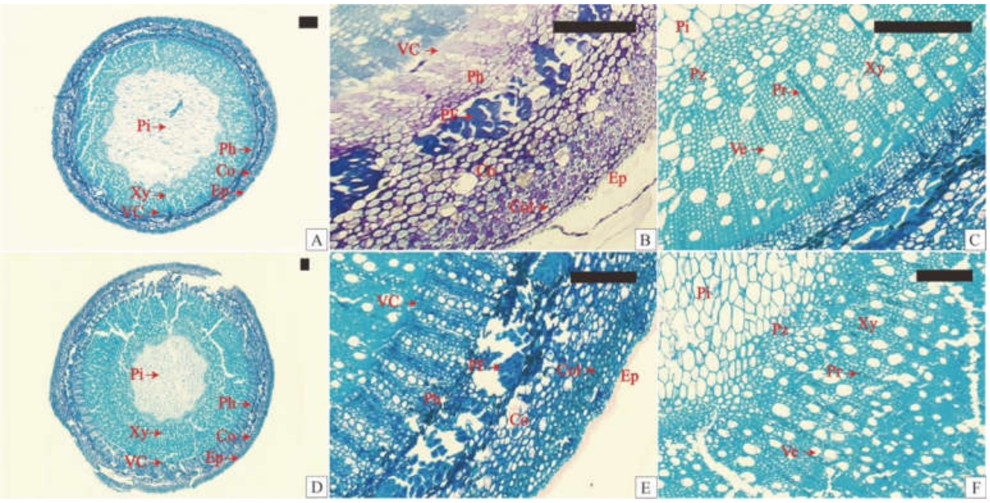

Figure S1. Cross-sectional anatomical structure of the current year's stems of GF and ZS.A-C show the anatomical structure of the current year's stems of the GF; Figures D-F show the anatomical structure of the current year's stems of the ZS. (A, D) The complete anatomical structure of the stem; (B, E) The anatomical structure of the stem, showing the epidermis, cortex, phloem, and vascular cambium; (C, F) The anatomical structure of the stem, showing the xylem and pith. In Figures A-F, the scale bar represents 200 μm. Co: Cortex; Col: Collenchyma; Ep: Epidermis; PF: Phloem Fibers; Ph: Phloem; Pi: Pith; Pr: Medullary Rays; Pz: Perimedullary Zone; VC: Vascular Cambium; Ve: Vessel; Xy: Xylem.

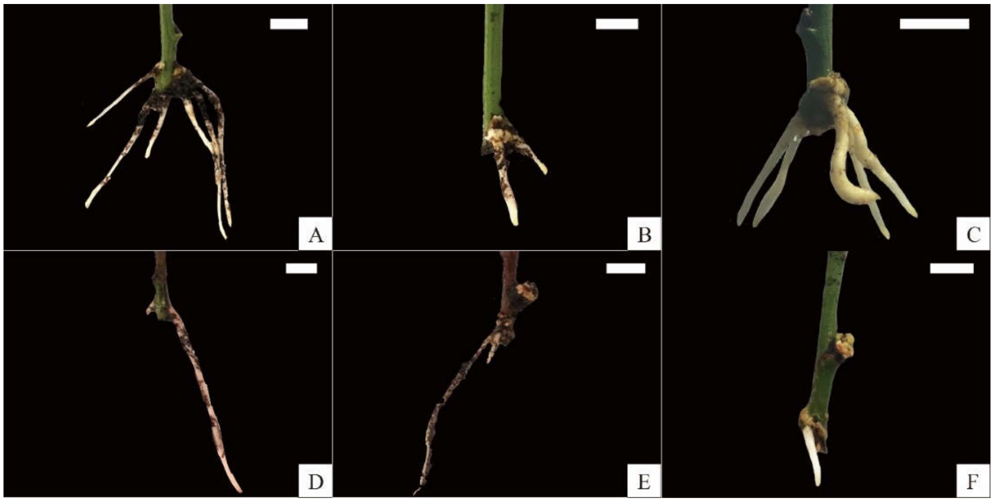

Figure S2. Different types of rooting of GF and ZS cuttings. A-C represent different types of root formation in cuttings of GF; Figures D-F represent different types of root formation in cuttings of ZS. (A, D) Adventitious roots extending from the lateral cortex of the stem; (B, E) Adventitious roots emerging from callus tissue; (C, F) Adventitious roots emerging from areas with less callus tissue at the lower cut of the cuttings. In Figures A-F, the scale bar represents 10 mm.

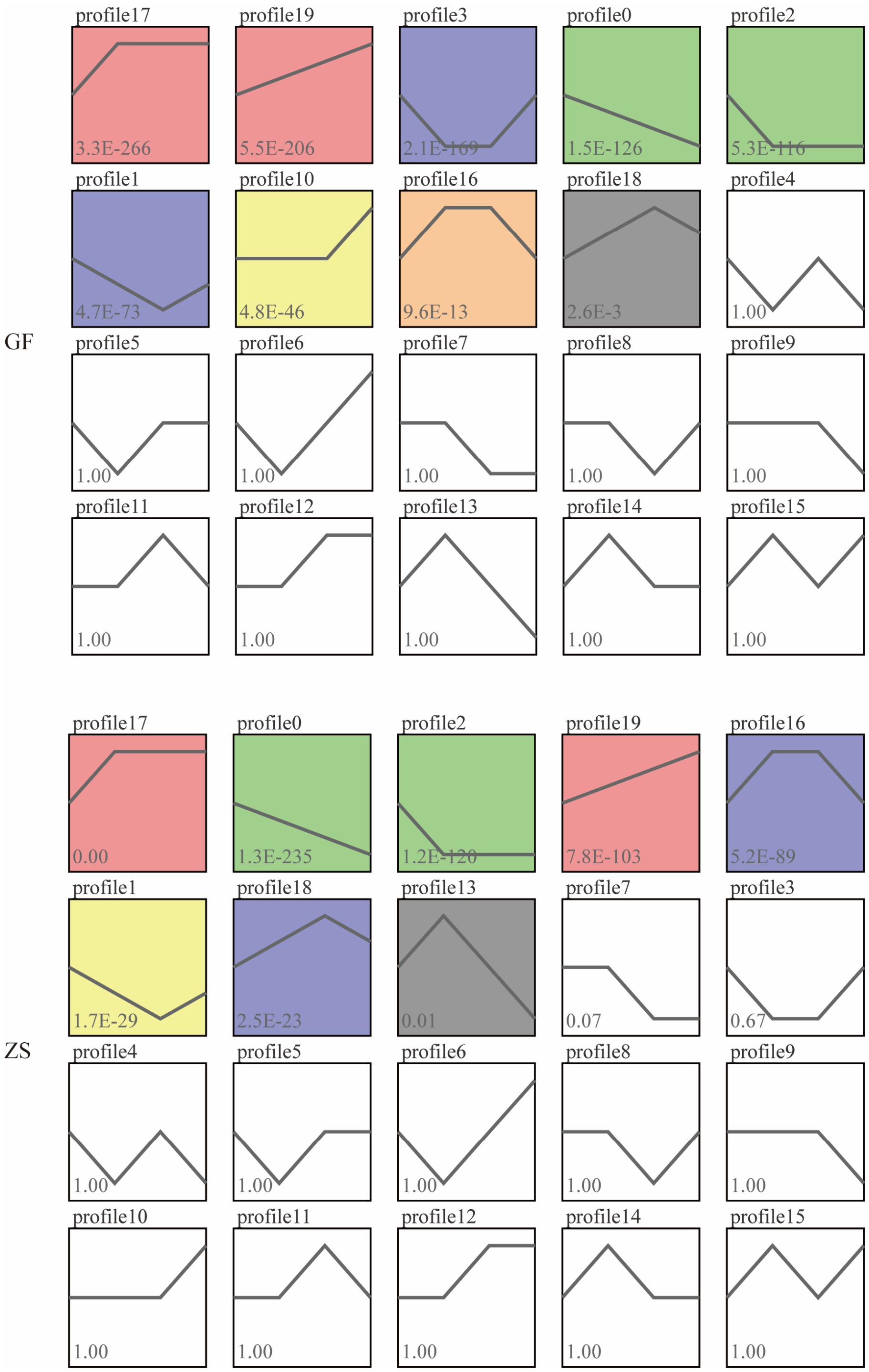

Figure S3. Gene expression trend analysis for GF and ZS. The colored background indicates highly significant correlation (p < 0.01), while the black and white background indicates non-significant correlation.

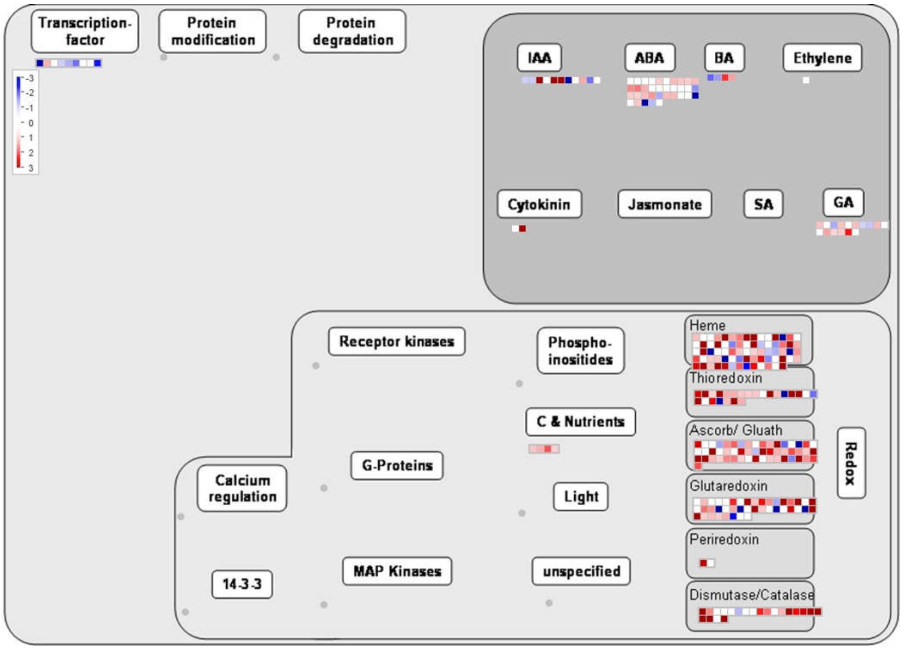

Figure S4. MapMan annotation of differentially expressed genes associated with hormone formation.

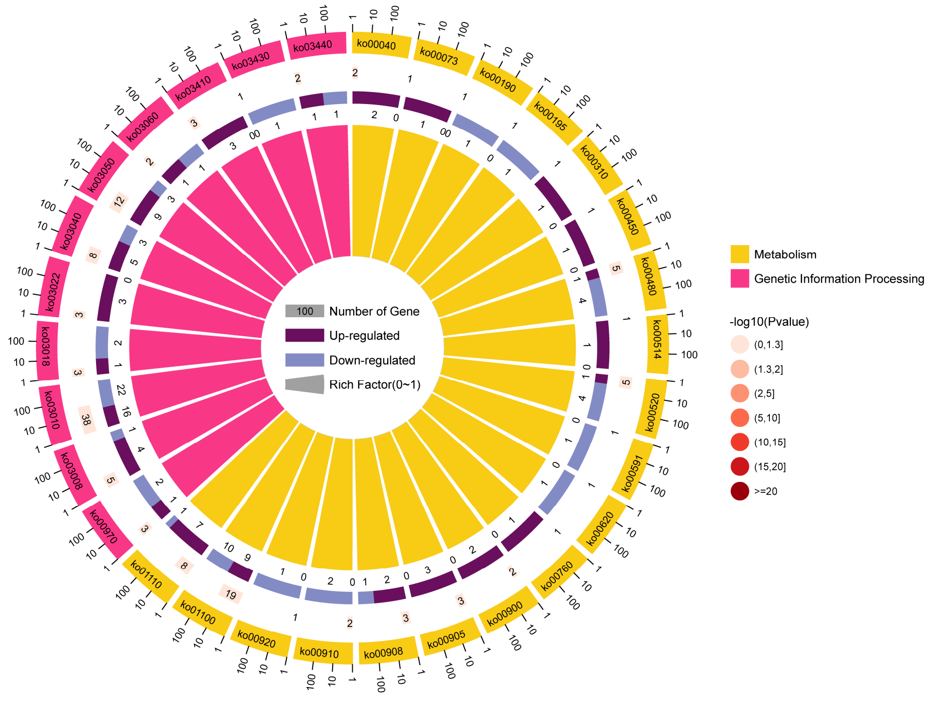

Figure S5. KEGG enrichment of consistently up-regulated differentially expressed genes.

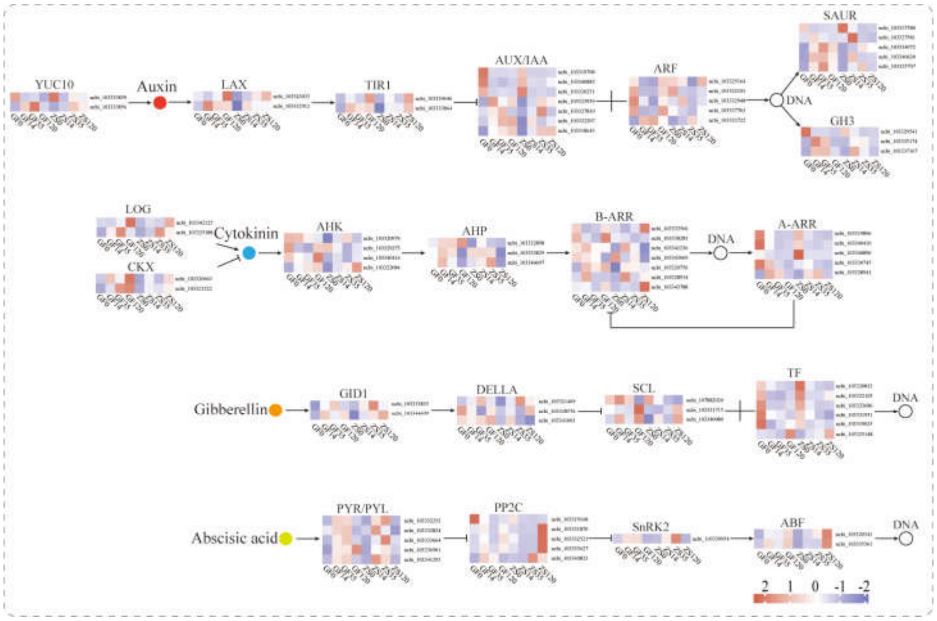

Figure S6. Expression profiles of phytohormone-related genes during the semi-softwood cutting process of *P. mume*. Red indicates up-regulated genes, and blue indicates down-regulated genes. 0d: original cuttings; 14d: cuttings at the initial expansion stage; 35d: cuttings at the callus formation stage; 120d: cuttings at the rooting stage.

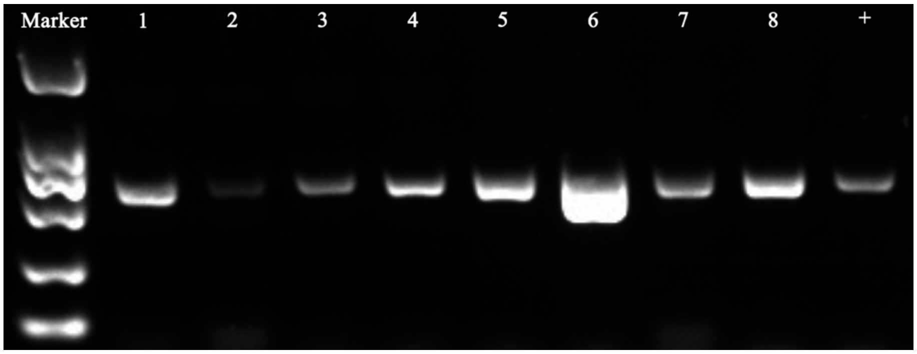

Figure S7. Identification of Transgenic Positive Seedlings. The molecular weight of the marker is 2000 base pairs (bp).

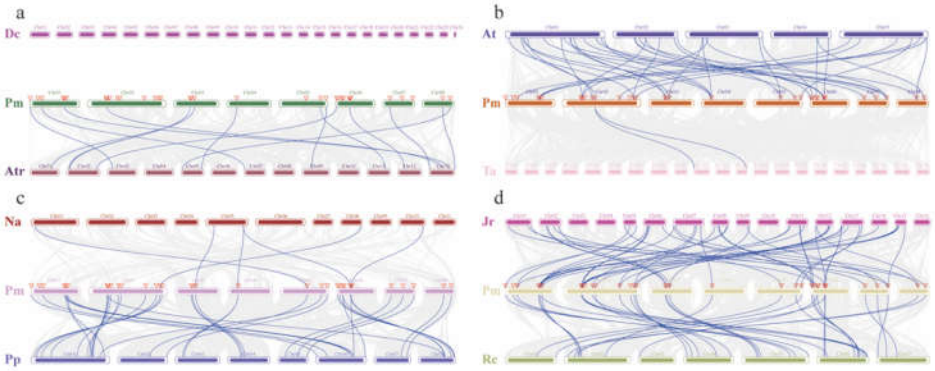

Figure S8. Macrosynteny pattern between the *P. mume* and other plants karyotype of genes associated with adventitious root formation. Grey lines highlight the syntenic blocks spanning the genome. Blue lines highlight the major segmental duplications of Genes inter-chromosomes. (a) *D. complanatum* (Dc) vs. *P. mume* (Pm) vs. *A. trichopoda* (Atr); (b) *T. aestivum* (Ta) vs. *P. mume* (Pm) vs. *A. thalina* (At); (c) *P. persica* (Pp) vs. *P. mume* (Pm) vs. *N. attenuata* (Na); (d) *R. chinensis* (Rc) vs. *P. mume* (Pm) vs. *J. regia* (Jr).
